# 4-nitroquinoline 1-oxide induces immune cells death to onset early immunosuppression during oral squamous cell carcinoma development

**DOI:** 10.3389/fimmu.2023.1274519

**Published:** 2023-10-23

**Authors:** Satya Ranjan Sahu, Shweta Thakur, Doureradjou Peroumal, Bhabasha Gyanadeep Utkalaja, Abinash Dutta, Premlata Kumari, Ipsita Subhadarsini, Narottam Acharya

**Affiliations:** ^1^ Laboratory of Genomic Instability and Diseases, Department of Infectious Disease Biology, Institute of Life Sciences, Bhubaneswar, India; ^2^ Regional Center of Biotechnology, Faridabad, India

**Keywords:** DNA damage response, carcinogenesis, cell death, p53, oral squamous cell carcinoma, cancer immunology, T cells, B cells

## Abstract

4-Nitroquinoline *N*-oxide (4-NQO) and its derivatives react with genomic DNA to form stable quinolone monoadducts, which are highly mutagenic and genotoxic. While the chronic high-dose exposure of epithelial cells to a carcinogen such as 4-NQO leads to tumor development, its effect on other cells has not been explored yet. Since the immunosuppression due to aberrant immunological profile is recognized as a significant cause in tumors, here we determine the interaction between 4-NQO and immune cells both *in vivo* and *in vitro*, and its effect on oral squamous cell carcinoma (OSCC) progression in a murine model. Immune cell profiling of the spleen and peripheral blood revealed a significant decrease in the B-cell population in 4-NQO-exposed mice than the untreated group. Additionally, γδ T and CD5^+^ B lymphocyte populations decreased at both pre- and post-cancerous stages of OSCC. These results suggested that 4-NQO induced tumor transition from pre-malignant lesions to OSCC by altering certain immune cells systemically. Next, to establish the effect of 4-NQO on immune cells, human B- and T-cell lines were subjected to 4-NQO; the reduction in cell viability, increase in DNA damage response marker, and induction of apoptosis were more pronounced in B than T cells. Altogether, our results indicated that in addition to the genotoxicity of oral epithelial cells, 4-NQO potentiates long-range effects on specific immune cells to induce cell death to cause very-early immunosuppressive response during oral carcinogenesis, and thus immunosuppression and tumor development are coevolved.

## Introduction

4-Nitroquinoline-1-oxide (4-NQO), a quinolone-derived synthetic molecule, is classified as a DNA-reactive genotoxin that induces bulky adducts in the genome. It is metabolized into a highly reactive 4-acetoxyaminoquinoline-1-oxide (Ac-4HAQO) that makes covalent adducts to *C* ^8^ or *N* ^2^ of deoxyguanosine and *N* ^6^ of deoxyadenosine in DNA. 4-NQO also produces oxidative damage, single DNA strand breaks, and irreversible DNA-protein crosslinks (1, 2). 8-Hydroxydeoxyguanosine (8OHdG) DNA lesions generated due to 4-NQO treatment, when it pairs with adenine and cytosine during replication, lead to an increase in G:C to T:A transversion mutations (3). Owing to its known mutagenic and potent carcinogenic properties, 4-NQO is frequently used in genotoxicity assays and specifically employed for developing carcinogen-induced oral squamous cell carcinoma (OSCC) mouse models, and its carcinogenic effect is comparable to tobacco carcinogen-induced genetic and molecular alterations (4–7). Such 4-NQO-mediated changes result in the transformation of murine epithelium into initial pre-cancerous lesions, and then cancerous lesions in the tongue and oral mucosa, simulating human oral carcinogenesis (8–10).

OSCC is a common type of malignancy in head and neck cancers (HNCs), representing as sixth most frequent cancer worldwide, with an overall 5-year survival rate of approximately 50% (11). Although both *in vitro* and *in vivo* models have been used for various cancer studies, the *in vivo* experiments using animal models especially the 4-nitroquinoline 1-oxide (4-NQO) mouse models are preferable because of their ability to capture the malignant transformation of early-stage lesions and mirror the interaction of the functional immune system with tumor, similar to reflecting an almost accurate tumor microenvironment in humans. Implementation of the 4-NQO-induced oral cancer mouse model is based on the premise that it predominantly induces oral cavity cancer by simulating the gradual progression from dysplasia to invasion as seen in OSCC patients (4, 12, 13). It was also reported that molecular events displayed by the 4-NQO-induced OSCC mouse model mimic the human HNSCC patients (14). Genetic alterations of 4-NQO-induced mice were investigated in lingual mucosa samples harvested from different stages, viz., normal tissue (0 weeks), early stage (12 weeks), and advanced stage (24 weeks), and using microarray and methylated DNA sequencing analysis; it was revealed that significant alterations took place in 63 hub genes as well as promoter methylation, and all of their human orthologous genes were found to be linked with human OSCC (15). Similarly, another study demonstrated that the immunopathology of murine oral tumor and human oral tumor are indistinguishable as determined by immunohistochemistry of cyclin D1 and E-cadherin labeling (16). Aberrant expressions of cell growth-related genes such as p-Erk1/2, Cox2 protein, and Rarβ2 in 4-NQO-induced murine oral and esophageal cancer tissue models were found to be very comparable to those in human esophageal cancer cell lines and patient tissue samples (8). Therefore, due to considerable pathological, morphological, genetic, and molecular analogies between the 4-NQO murine model and human OSCC, this model remains an ideal choice to understand the pathogenesis mechanism of OSCC and to evaluate the efficacy of different cancer prevention agents *in vivo*.

In current days, a speculative framework has emerged to understand the role of immune response to cancer and the factors that affect the success of immune-based cancer therapies. Immune system has a tendency to remove nascent premalignant cells and prevent tumor development and progression; accordingly, any derangement in the immune system may allow cancer cells to thrive. OSCC is often characterized by a state of acute immunosuppression, displaying low lymphocyte counts and impaired T-cell and NK-cell activities (17). Elevated expression of proinflammatory genes like s100a9, IL23a, IL1b, and immune checkpoint gene PDCD1/PD1 was reported during oral cancer development in the 4-NQO–induced cancer group in comparison to normal mice, and similar results have also been found in human oral cancer and other cancers (18, 19). Although it is not clear whether the alteration in immune response is a cause or effect of the neoplasm, unlike in individuals with adenocarcinomas, melanomas, or sarcomas, these responses remain compromised even after the surgical treatment of the tumor in patients with head and neck cancer. Thus, it appears that the immune defect in head and neck cancer could be a primary event. Therefore, a comprehensive understanding of the dysregulation of immune system from pre-cancerous to cancer stages is required to determine the role of immune cells in oncogenesis, design advanced immuno-therapeutic strategies, and improve outcomes for patients with OSCC. Although 4-NQO has been reported to be immunosuppressive at certain concentrations, there still remains a gap in knowledge on how carcinogenic 4-NQO modulates the immune response that impacts tumor progression (20). Therefore, here by using a classical 4-NQO-induced OSCC murine model, we demonstrate a modulatory impact of 4-NQO on immune cells in pre-malignant to malignant transformation to cause systemic immune suppression that was reflected mostly by reduced B-lymphocyte and γδ T-cell distribution in spleen and circulating blood, thereby suggesting a different strategy for consideration of advanced immunomodulatory therapeutic options for OSCC.

## Results

### Development of 4-NQO-induced pre- and post-OSCC models

**Figure 1:**
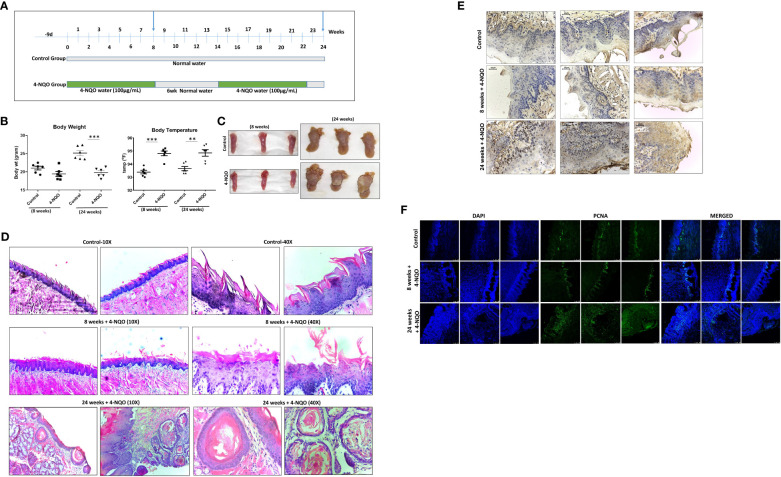
4-NQO–induced oral carcinogenesis murine models and their characterization. **(A)** A schematic representation of the experimental design to generate mouse models of 4-NQO-induced oral carcinogenesis (n = 6) at 8 and 24 weeks. **(B)** Measurement of weights and body temperature of animals prior to their sacrifice at 8 and 24 weeks. Mean ± SD, **P-value <0.01 and ***P-value <0.001. **(C)** Representative photograph of individual tongues of mice (n = 3) treated with or without 4-NQO at 8 and 24 weeks. **(D)** Histopathological analysis and representative microscopic images of tumor area using hematoxylin and eosin (H&E) staining of mouse tongues with and without 4-NQO treatment (n = 2) at 8 and 24 weeks at 10× and 40× resolution. **(E)** Immunohistochemistry analysis for p53 expression in the tongues of control and pre- and post-OSCCs mice groups induced by 4-NQO (n = 3) at 8 and 24 weeks. **(F)** Immunofluorescence analysis of proliferation marker PCNA expression in the tongues of control and pre- and post-OSCCs mouse groups induced by 4-NQO (n = 3) at 8 and 24 weeks.

Chronic exposure to 4-NQO in drinking water for a period of 20 to 24 weeks is sufficient to develop OSCC in mice. Although 4-NQO molecules remain in the systemic circulation, within this period, not any other tissues are reported to develop cancerous lesions in mice. To determine the effect of 4-NQO during OSCC progression, we wanted to compare immune cell profiles at pre- and post-OSCC stages. To obtain such experimental model systems (n = 6 for each group), one group of mice was provided 4-NQO for 8 weeks continuously, and for the other group, 4-NQO was provided twice of 8-week period and 6-week period of normal water in between. Two groups of control mice were also obtained of similar durations without any 4-NQO exposure (**Figure 1A**). Prior to their sacrifice at 8 weeks and 24 weeks, the weight and body temperatures of the animals were recorded, and blood was collected. After sacrificing the animals, tongue and spleen organs were excised and imaged. A significant reduction in body weight with hyperthermia was observed in animals exposed to 4-NQO than in the control groups (**Figure 1B**). The control group mice gained marginal body weight at 24 weeks in comparison to 8 weeks (~21 g vs ~25 g). At 8 weeks, the tongues of both the groups of mice were visibly very similar except few leucoplakia lesions in the 4-NQO treatment group mice, whereas tumors were apparently visible only on the tongues of 24-week carcinogen-treated mice (**Figure 1C**). Premalignant lesions found on the tongue at 8 weeks could be the precursors to oral cavity cancers in 24-week mice; for confirmation, histological staining with H&E was carried out (**Figure 1D**). The macroscopic and histopathological features of the tongue were observed to be normal in the control groups. Over time, the 4-NQO-treated group displayed histological changes from normal mucosa to abnormal hyperplasia at 8 weeks, and eventually invasive carcinoma in 24 weeks. The disintegration of the papillary layer was apparently visible in 4-NQO-treated mice. Further confirmation of pre-OSCC (8 weeks) and post-OSCC (24 weeks) stages was carried out by examining the presence of prognostic marker p53 and proliferation marker PCNA (**Figures 1E, F**). A high frequency of mutation in the TP53 gene that encodes for p53 tumor suppressor and upregulation of mutant p53 protein has been reported in a variety of human tumors (21). Due to the short half-life (20 min), a barely detectable concentration of wild-type p53 protein is typically found in normal cells. However, due to acquired mutations in p53 or its degradation pathways, the half-life of p53 gets extended; thereby, its concentration increases in tumors. PCNA, an essential homo trimeric sliding clamp, functions as a docking platform for the components of the eukaryotic DNA replication and DNA repair machineries (22, 23). While it is required for normal cellular growth, cell cycle progression, and differentiation, its expression is found to be upregulated in most cancerous cells including OSCC (24). Consistent with the reported data, the post-OSCC group displayed prominent immune reactivity to both PCNA (**Figure 1F**) and p53 (**Figure 1E**) antibodies in comparison to pre-OSCC and control groups.

### Splenic distribution of myeloid granulocytes, non-granulocytes, and lymphoid cells during OSCC development

**Figure 2:**
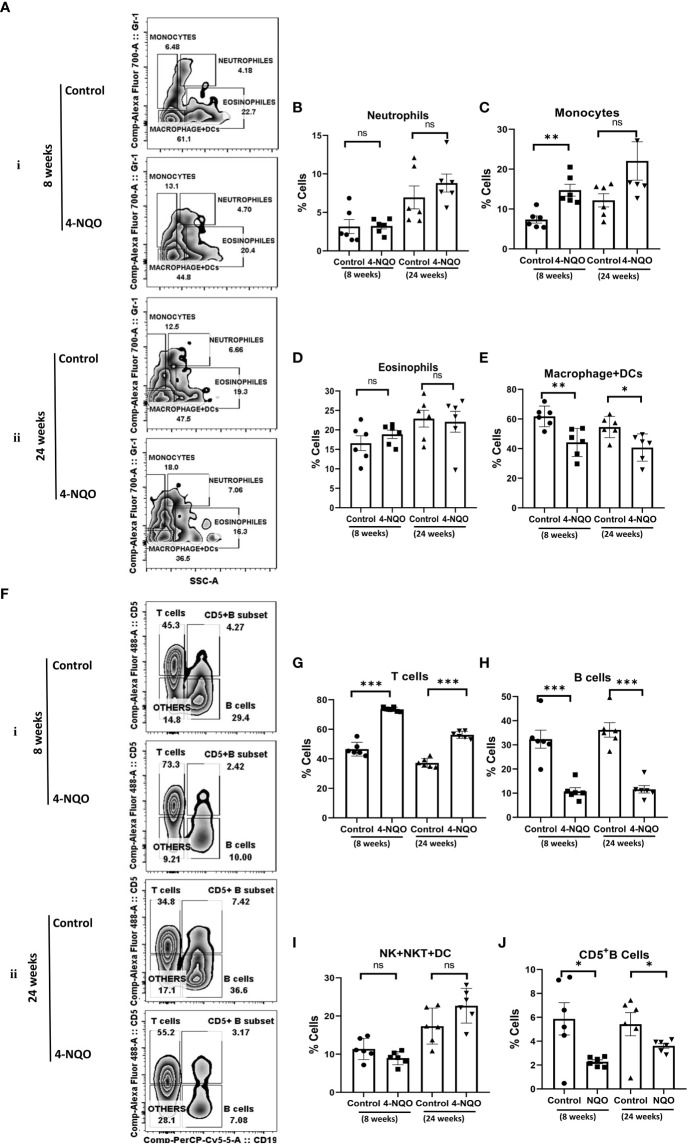
Splenic distribution of myeloid and lymphoid cells. **(A)** A representative bivariate density plot of Gr-1(Ly-6G/Ly-6C) (y-axis) vs side scatter (x-axis) for the analysis of the compartmental distribution of CD11b^+^ cells in the spleen of mice with and without 4-NQO exposure at 8 (i) and 24 (ii) weeks using FlowJo Software v8.0.2. Mean and standard error of the mean of percent total of **(B)** CD11b^+^/Gr-1^+hi^/SSC^(hi)^ neutrophils, **(C)** CD11b^+^/Gr-1^+^/SSC^(lo)^ monocytes, **(D)** CD11b^+^/Gr-1^-/lo^/SSC^(hi)^ eosinophils, and **(E)** CD11b^+^/Gr-1^−^/SSC^(lo)^ myeloid DCs and macrophages. **(F)** A representative bivariate density plot of CD5 (y-axis) vs CD19 (x-axis) for the analysis of the compartmental distribution lymphoid cells in the spleen of mice with and without 4-NQO exposure at 8 (i) and 24 (ii) weeks using FlowJo Software v8.0.2. Mean and standard error of the mean of percent total of **(G)** CD11b^−^/CD19^−^/CD5^+^ T cells, **(H)** CD11b^−^/CD5^+/−^/CD19^+^ B cells, **(I)** CD11b^−^/CD5^−^/CD19^−^ other lymphoid cells such as NK, NKT, lymphoid DCs, and **(J)** as CD5^+^ B cells. Mean ± SD, ns, non-significant, *P-value <0.05, **P-value <0.01, and ***P-value <0.001.

OSCCs are highly immunogenic tumors that are often characterized by abundant infiltration of macrophages, DC, mast cells, and lymphocytes, and some of these cells have been considered as prognostic markers for the survival of cancer patients. Among them, the M2 macrophages identified by CD163 and mature NK cells identified by CD57 are the most promising prognostic immune cells (17, 25). These immune cells’ distribution in tumors is most likely a secondary response to a neoplasm. Since 4-NQO was provided to animals in drinking water, we argued that in addition to its effect on epithelial cells of the oral cavity, it may affect both circulating and secondary lymphoid organ-resident immune cells. Therefore, the splenic distribution of immune cells was examined in harvested spleen from the animals with pre-OSCC and post-OSCC lesions. Using a multicolor immunophenotyping approach, various immune cell distributions were evaluated by flow cytometry (**Figure 2**). A gating strategy of such analyses is represented in supporting **Figure 1A**. We did not observe any significant change in the size of spleens of animals at the 8-week stage; however, although the overall size of spleens was larger in older mice, a noticeable size reduction was observed in post-OSCC models due to 4-NQO exposure (**Figure S2**). 4-NQO had minimal effect on granulocytes as the splenic neutrophil (CD11b^+^/Gr-1^+/hi^/SSC^(hi)^) and eosinophil (CD11b^+^/Gr-1^−/lo^/SSC^(hi)^) distribution in animals remained about the same in both pre- and post-OSCC groups when compared with the respective controls (**Figures 2A, B, D**). However, an increase in inflammatory monocytes (CD11b^+^/Gr-1^+^/SSC^(lo)^) but a reduction in myeloid DCs & macrophages (CD11b^+^/Gr-1^-^/SSC^(lo^) populations was observed in both OSCC groups than those in corresponding control spleens (**Figures 2A, C, E**).

**Figure 3:**
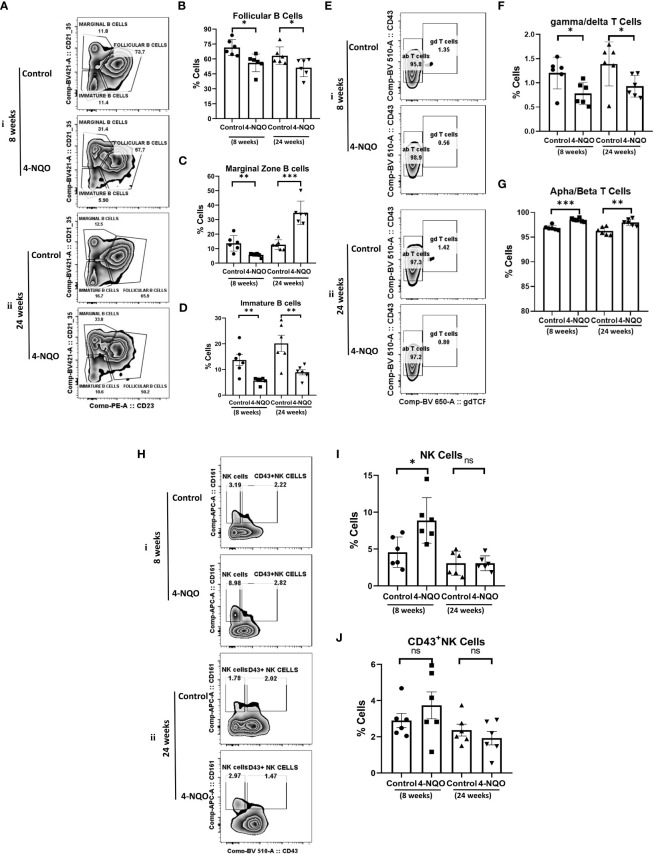
Splenic distribution of B-, T- and NK-cell subpopulations. **(A)** A representative bivariate density plot of CD21 (y-axis) vs CD23 (x-axis) for the analysis of the compartmental distribution of B cells in the spleen of mice with and without 4-NQO exposure at 8 (i) and 24 (ii) weeks using FlowJo Software v8.0.2. Mean and standard error of the mean of percent total of **(B)** CD11b^−^/CD5^−^/CD19^+^/CD21^+Mid^/CD23^+^ follicular B cells **(C)** CD11b^−^/CD5^−^/CD19^+^/CD21^+hi^/CD23^−^ marginal zone B cells and **(D)** CD11b^−^/CD5^−^/CD19^+^/CD21^−^/CD23^–^ immature B and B1a cells**. (E)** A representative bivariate density plot of CD43 (y-axis) vs γδTCR (x-axis) for the analysis of the compartmental distribution of T cells in the spleen of mice with and without 4-NQO exposure at 8 (i) and 24 (ii) weeks using FlowJo Software v8.0.2. Mean and standard error of the mean of percent total of **(F)** CD11b^−^/CD19^−^/CD5^+^/CD43^+^/γδ-TCR^+^ gamma/delta-T cells and **(G)** CD11b^−^/CD19^−^/CD5^+^/CD43^+^/γδ-TCR^−^ alpha/beta-T cells. **(H)** A representative bivariate density plot of CD161 (y-axis) vs CD43 (x-axis) for the analysis of the compartmental distribution of NK cells in the spleen of mice with and without 4-NQO exposure at 8 (i) and 24 (ii) weeks using FlowJo Software v8.0.2. Mean and standard error of the mean of percent total of **(I)** CD11b^−^/CD5^−^/CD19^−^/CD161^+^/CD43^−^ NK cells and **(J),** CD11b^−^/CD5^−^/CD19^−^/CD161^+^/CD43^+^ NK cells. Mean ± SD, ns, non-significant, *P-value <0.05, **P-value <0.01, and ***P-value <0.001.

The analysis of lymphoid cells revealed that the proportion of T cells (CD11b^−^/CD19^−^/CD5^+^) was higher in both 4-NQO treatment groups than in respective control groups, contrary to the proportion of total B cells (CD11b^-^/CD19^+^/CD5^+/−^) which were significantly low in OSCC groups irrespective of stages as compared to control (**Figures 2F–H**). However, the proportion of other lymphoid cells such as NK, NKT, and lymphoid DCs (CD11b^−^/CD5^−^/CD19^−^) remained unaltered between control and 4-NQO-treated spleens (**Figures 2F, I**). Again, among the B-cell subtypes, we found reduced populations of CD5^+^, follicular (CD11b^-^/CD5^-^/CD19^+^/CD21^+Mid^/CD23^+^), and immature B cells (CD11b^−^/CD5^−^/CD19^+^/CD21^−^/CD23^–^) in 4-NQO-treated groups, but the marginal-zone B-cell (CD11b^−^/CD5^−^/CD19^+^/CD21^+hi^/CD23^−^) population varied differently in OSCC models (**Figures 2F, J**, **Figures 3A–D**). While a reduced population was seen in precancerous stages, they increased drastically in post-OSCC animals than the controls. The gamma-delta (γδ) and alpha-beta (αβ) T cells are two different T-cell lineages that differ by receptor expressions. Gamma-delta (γδ) T cells (CD11b^−^/CD19^−^/CD5^+^/CD43^+^/γδ-T CR^+^), which are known for their inflammatory response by producing several cytokines like GM-CSF, IL-4, IL-17, IL-21, IL-22, and IFN-γ, were decreased in the 4-NQO group of both pre- and post-OSCC, whereas the population of αβ-T cells (CD11b^−^/CD19^−^/CD5^+^/CD43^+^/γδ-T CR^−^) was somewhat increased (**Figures 3E–G**). Interestingly, CD43-positive and -negative NK cells (CD11b^−^/CD5^−^/CD19^−^/CD161^+^/CD43^−/+^) increased only at the precancerous stage (**Figures 3H–J**). These results suggested that there were marked differences in the tissue-resident immunological profile between the control group and 4-NQO-treated pre-OSCC and post-OSCC mice. Thus, immune cell derangement is not a post-cancerous event caused by 4-NQO; rather, it initiates at a very early stage of cancer progression that persists till tumor development and beyond.

### Impact of 4-NQO on the circulating myeloid and lymphoid cell populations in blood

**Figure 4:**
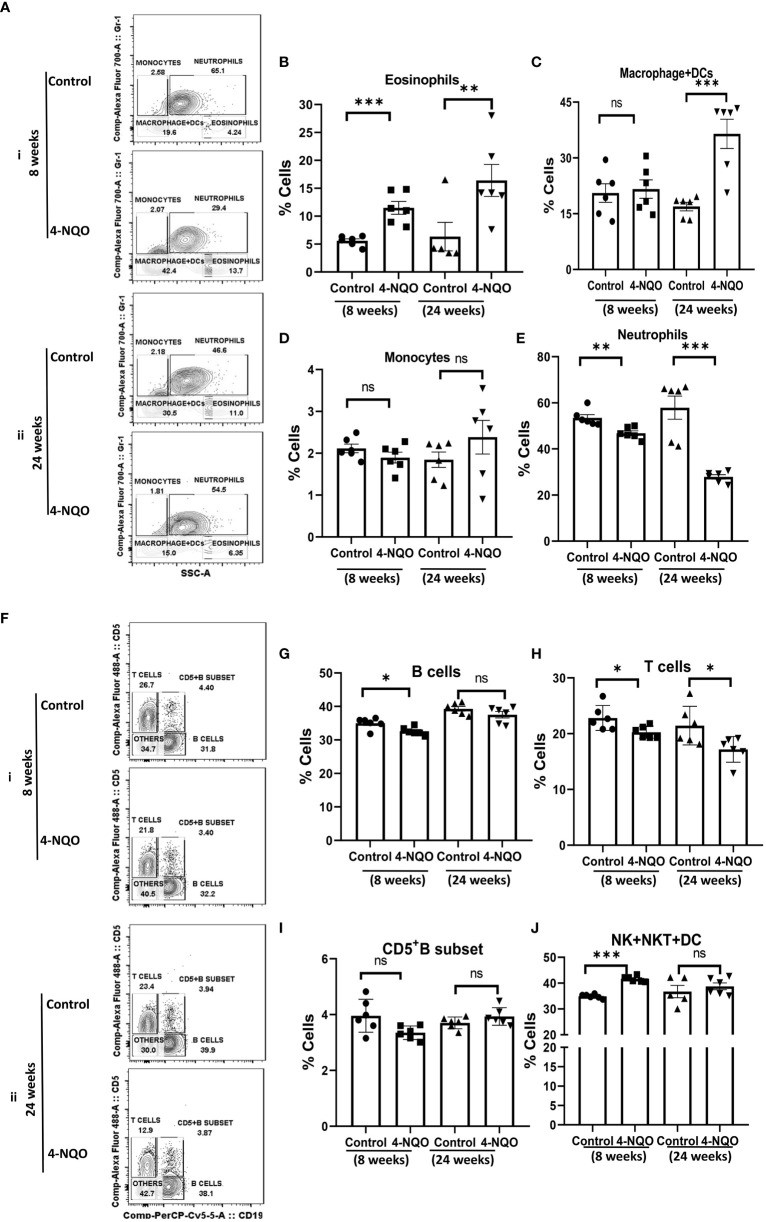
Circulating myeloid and lymphoid cell population in the blood. **(A)** A representative bivariate density plot of Gr-1(Ly-6G/Ly-6C) (y-axis) vs side scatter (x-axis) for the analysis of the compartmental distribution of CD11b^+^ cells in the blood of mice with and without 4-NQO exposure at 8 (i) and 24 (ii) weeks using FlowJo software v8.0.2. Mean and standard error of mean of percent total of **(B)** CD11b^+^/Gr-1^-/lo^/SSC^(hi)^ eosinophils, **(C)** CD11b^+^/Gr-1^−^/SSC^(lo)^ myeloid DCs and macrophages, **(D)** CD11b^+^/Gr-1^+^/SSC^(lo)^ monocytes, and **(E)** CD11b^+^/Gr-1^+hi^/SSC^(hi)^ neutrophils. **(F)** A representative bivariate density plot of CD5 (y-axis) vs CD19 (x-axis) for the analysis of compartmental distribution lymphoid cells in the blood of mice with and without 4-NQO exposure at 8 (i) and 24 (ii) weeks using FlowJo Software v8.0.2. Mean and standard error of the mean of percent total of **(G)** CD11b^−^/CD5^+/−^/CD19^+^ B cells, **(H)** CD11b^-^/CD19^−^/CD5^+^ T cells, **(I)** as CD5^+^ B cells, and **(J)** CD11b^−^/CD5^−^/CD19^−^ other lymphoid cells such as NK, NKT, and lymphoid DCs. Mean ± SD, ns, non-significant, *P-value <0.05, **P-value <0.01, and ***P-value <0.001.

Next, we assessed the changes in myeloid cell and lymphocyte subpopulations in the peripheral blood by using a similar immunophenotyping assay and gating strategy (**Figure S1B**). Unlike in the spleen, the percentage of eosinophils increased but no change in monocytes was detected in both early and late stages of OSCC. While the neutrophil population was reduced in 4-NQO-treated animals, macrophage + DC count was significantly higher in post-OSCC models when compared with the control and pre-OSCC groups (**Figures 4A–E**). Interestingly, most of the circulating lymphocyte populations (B, T, and CD5^+^B cells) were reduced at an early stage of OSCC development, but later on, their counts did not differ significantly between control and post-OSCC animals (**Figures 4F–I**). However, the 4-NQO-induced pre-OSCC group exhibited elevated levels of NK, NKT, and lymphoid DCs (CD11b^−^/CD5^−^/CD19^−^), with no significant difference in the post-OSCC group (**Figures 4F, J** **).** These results suggested that while the lymphoid populations reduced at the pre-OSCC stage, certain myeloid cells increased at post-OSCC conditions. Nevertheless, similar to splenic, the circulating immune cell populations also got altered upon 4-NQO treatment, and a reduction in both B and T cells was observed.

### Cytotoxic and genotoxic effect of 4-NQO on B and T lymphocytes

**Figure 5:**
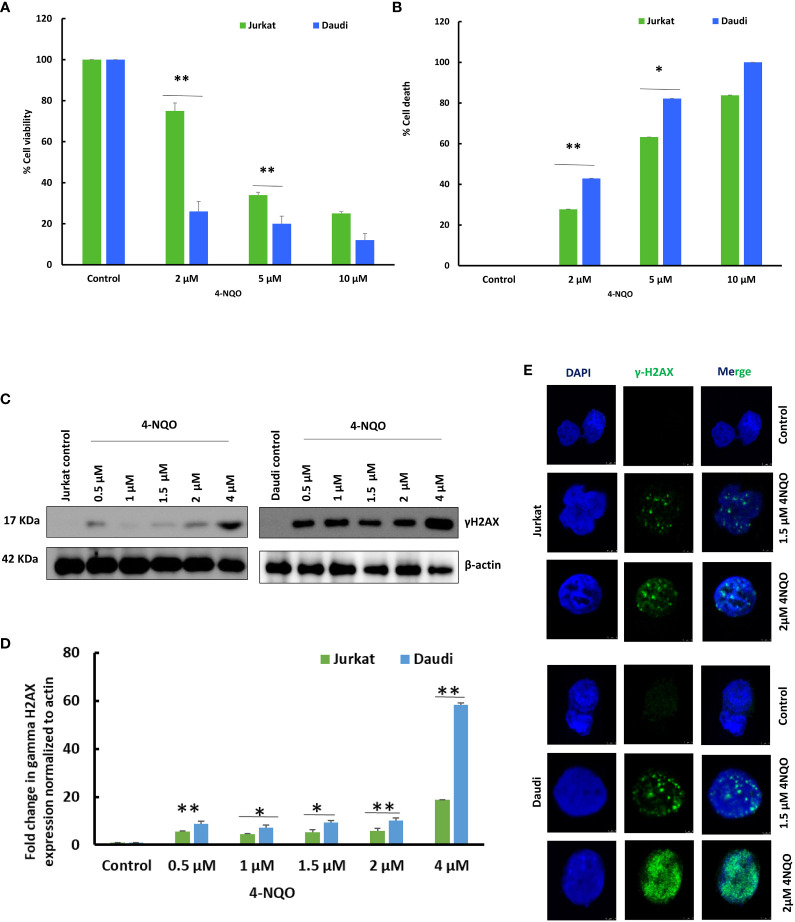
Effect of 4-NQO on Jurkat and Daudi cells. **(A)** Determination of cell viability of Jurkat and Daudi cells by MTS assay following treatment with 4-NQO (0, 2, 5, and 10 μM) in 48-well plates. **(B)** Determination of cell death of Jurkat and Daudi cells by trypan blue assay following treatment with 4-NQO (0, 2, 5, and 10 μM) in six-well plates. Data presented is derived from three individual experiments, and error bars are indicated. **(C)** Jurkat and Daudi cells were treated with different concentrations of 4-NQO at 0, 0.5, 1, 1.5, 2, and 4 μM individually for 48 h, and γH2AX expression was determined. **(D)** Densitometric analysis of the γH2AX blots when normalized to β-actin endogenous control. **(E)** Immunofluorescence analysis of γH2AX in 1.5 and 2 μM 4-NQO-treated Jurkat and Daudi cells. The experiments were repeated twice. Mean ± SD, ns, non-significant, *P-value <0.05 and **P-value <0.01.

Since we observed a reduced population of B and T cells in OSCC preclinical models, to understand the possible reasons for such an effect of 4-NQO, we further assessed the cell viability of Jurkat T and Daudi B transformed human cells in response to 4-NQO by using MTS and trypan blue exclusion assay (**Figures 5A, B**). Metabolically active cells produce a NAD(P)H-dependent dehydrogenase enzyme that reacts with MTS tetrazolium to produce purple-colored compounds which can be measured at 490–500 nm, whereas in trypan blue exclusion assay, upon addition of the dye, while the viable cells retain a clear cytoplasm, the cytoplasm of nonviable cells become blue in color. We observed that 4-NQO induces cytotoxicity in both Jurkat and Daudi cells in a dose-dependent manner; however, Daudi cells exhibited high susceptibility to 4-NQO as more reduction of viable cells were observed as compared to Jurkat cells as determined by both assays. About 2–5 µM of 4-NQO addition resulted in a significant increase in cell death in Daudi than in Jurkat cells.

Next, we investigated whether this observed reduction in cell viability in B cells is due to the result of any excessive DNA damage upon 4-NQO exposure, and the γH2AX expression level was validated (**Figures 5C, D**). γH2AX is an early molecular marker of DNA double-strand breaks (DSBs), as histone variant H2AX becomes rapidly phosphorylated at Ser-139 in response to DSBs. We found that the treatment with 4-NQO led to the activation of γH2AX in both cell lines as low as 4 μM concentration. In Jurkat cells, a modest increase in γH2AX expression was observed at all concentrations of 4-NQO; however, it increased significantly by several folds in Daudi cells. Furthermore, this result was confirmed by immunofluorescence detection of γH2AX foci in both cell lines that correlated with the expression analyses, and more γH2AX foci were detected in the nucleus of Daudi than Jurkat cells upon 4-NQO treatment (**Figure 5E**).

**Figure 6:**
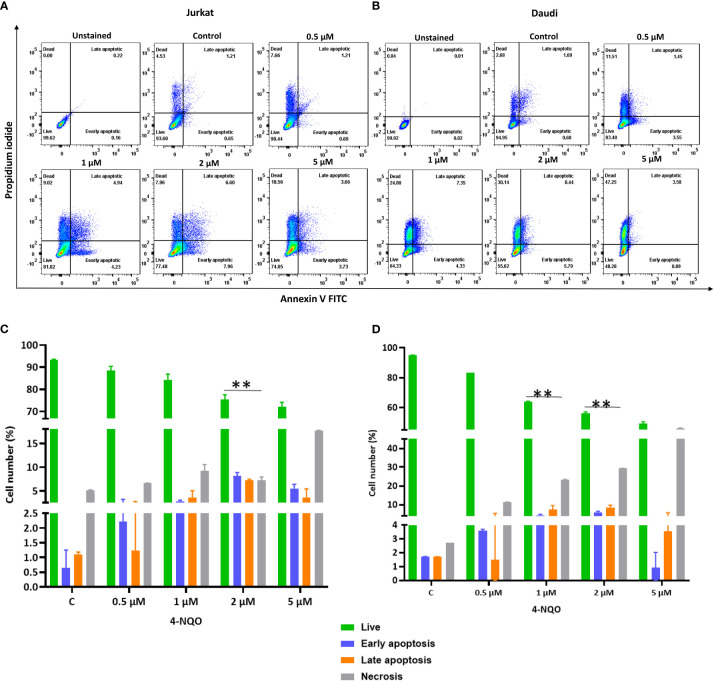
Flow cytometric cell death analysis of Jurkat and Daudi cells following 4-NQO treatment. **(A)** Jurkat and, **(B)** Daudi cells were grown in the presence of 4-NQO (0.5, 1, 2, and 5 μM) for 48 h and were stained with Annexin V/PI for detection of live (unstained), early apoptotic (Annexin V positive/PI negative), late apoptotic (Annexin V positive/PI positive) and necrotic cells (Annexin V negative/PI positive). The flow cytometry profile represents Annexin V-FITC staining in the x-axis and propidium iodide (PI) in the y-axis, dual staining of cells with Annexin V-FITC and PI categorizes cells into four regions, viz., Q1 (live cells), Q2 (early apoptotic cells), Q3 (late apoptotic cells), and Q4 (necrotic cells). Quantification of live, early- and late apoptotic, and necrotic cells of Jurkat **(C)** and Daudi cell lines **(D)**. Bar graph represents the means ± SEM of the cell percentage derived from two independent experiments. * p<0.05, and ** p<0.01.

Apoptosis is a secondary response to DNA damage. Stalling of replication fork due to excessive DNA breaks leads to cell death. Since we observed accumulation of γH2AX foci in both the cell lines, in order to assess the ability of 4-NQO to evoke apoptosis, flow cytometric analysis of Annexin V-FITC-PI-labeled Jurkat and Daudi cells was conducted (**Figures 6A–D**). With the increasing concentrations of 4-NQO, the percentage of apoptotic cells (early, late, and dead) was also increased for both the lymphocytic cell lines; however, consistent with our other results, here also Daudi cells exhibited a higher level of cell death than Jurkat cells at all the concentrations treated.

## Discussion

The immune system plays a critical role in monitoring tissue homeostasis, removing damaged cells, and preventing infections due to invading pathogens. Despite such an efficient surveillance system, cancers develop in humans with a high frequency. Recent insights from clinical studies and experimental preclinical models of carcinogenesis suggest a convoluted association of immune cells with developing tumors, and ultimately, certain immune suppression mechanisms adopted by the tumors allow them to thrive. Immune suppression by tumors is achieved in two ways: (a) by suppressing MHC expression and release of anti-inflammatory immunosuppressive cytokines like Il-10, Il-1b, TGF-β, and prostaglandin E2, etc., and (b) by altering the host’s immune cell population (26–28). Numerous studies have evaluated tumor-infiltrated, peripheral, and other lymphoid organs’ specific immune cell representation in different types of neoplasms, and some reports suggest their possible prognostic role (25, 29). Since most of these immunological analyses were carried out after tumor development, the demonstrated immunosuppression is most likely a deleterious consequence of advanced tumor, although the mechanism underlying tumor-associated immune suppression is yet to be fully elucidated. Therefore, this study was planned to determine any alteration in immune cell profiling during the onset of oncogenesis and compare it with that which occurs post-tumor development, and secondly, to determine the effect of a carcinogen on immune cells that may also induce immune suppression. Since the murine model of 4-nitroquinoline 1-oxide (4-NQO)-induced OSCC is a near mimic of human OSCC but provides an opportunity to understand cancer progression at very early to late stages, we took advantage of this popular OSCC model to understand the direct and indirect effects of 4-NQO on immune cells.

First, we ascertained the development of 4-NQO-induced premalignant lesions predominantly on the tongue around the 8th week of 4-NQO administration via drinking water, which progressed to OSCC at the 24th week by H&E staining and verifying the expression status of well-known proliferative and suppressors markers of OSCC. The pre-OSCC mice showed the presence of low-grade epithelial dysplasia. Even after switching to normal water for 6 weeks in between followed by 4-NQO till the 24th week, the lesions continued to advance into OSCC, suggesting 4-NQO’s role in the initiation of cancer and the underlying molecular changes thereafter. The duration of tumor development and morphological changes of OSCC lesions were very similar to earlier reported studies (30–33). Differential staining patterns for PCNA and p53 were observed between pre-OSCC and post-OSCC groups when compared with normal control. Studies have shown that the expression of PCNA can be used to characterize the malignant potential of oral cavity lesions (34, 35), and we observed increased expression of PCNA in tongue tissues of the post-OSCC group, advocating for the role of PCNA as a marker in characterizing normal, hyperplastic, and dysplastic epithelia in the oral cavity. Overexpression of the p53 tumor suppressor in post-OSCC models suggests it is likely to be a non-functional protein.

Immune surveillance is often found in the early stage of OSCC (36). It has been suggested that the precancerous oral lesion has a major immunological impact within the premalignant lesion, throughout the oral cavity, in lymph nodes, and in circulation (37). Both macrophages and neutrophils are known as the first-line responders against injury or infection. Understanding the role of tumor-associated macrophages (TAMs) and tumor-associated neutrophils (TANs) remains ambiguous because switching into activation states with both pro- and antitumor properties as well as identifying their population based on specific surface marker expression is tough due to the lack of specific markers (38). Studies showed that the main infiltrating cells in the 4-NQO-induced murine and rat model were CD11b^+^ macrophages (39, 40). In our study, we found an increase in splenic CD11b^+^ monocytes in the pre-OSCC group but a decrease in CD11b^+^ macrophages in both the pre- and post-OSCC groups. The peripheral representation of these immune cells did not alter except for an increased population of CD11b^+^ macrophages in the post-OSCC model. Whether this tissue-specific differential distribution of monocytes and macrophages has any role in immune suppression requires further investigation. Hypereosinophilia was prominently observed in both tissues and stages of OSCC progression. Lymphocytes are the main immune effectors in head and neck squamous cell carcinoma. Changes in peripheral blood lymphocyte subtypes have been described as related to cytotoxic T lymphocytes, helper T lymphocytes, and regulatory T cells during the development of oral and maxillofacial squamous cell carcinoma (41–43). Earlier studies have demonstrated that the inflammatory infiltrate cell population increases in the human tongue OSCC while premalignant lesions transform into developed tumors. A related study showed an increase of CD4^+^, CD8^+^, and CD19^+^ lymphocyte infiltration in dysplastic lesions in comparison to normal epithelium suggesting elevated T- and B-cell response in dysplastic lesions (44) whereas another study reported a decrease in plasma cells and other B-lymphocytes to be associated with high-grade dysplastic lesions and less differentiated OSCC (45). Also observed is an elevation in the proportion of CD4^+^ and CD8^+^ T cells in mice with premalignant lesions as compared to the healthy control and HNSCC-bearing groups (33). Zhao et al. found a gradual increase in the frequency of CD4^+^CD25^+^ FoxP3^+^ T_regs_ in the peripheral blood and regional lymph nodes of 4-NQO-induced rat tongue carcinogenesis during disease progression (46). The pre-therapeutic human blood analysis data showed a lower proportion of helper T lymphocytes (CD4^+^), a significantly higher level of cytotoxic/suppressive T lymphocytes (CD8^+^), and a much lower CD4^+^ T lymphocyte/CD8^+^ T lymphocyte ratio compared to control subjects. Conversely, the evaluation of circulating NK (CD16+) cells showed a markedly higher pre-therapeutic level compared to the control group (29). Furthermore, the level of B lymphocytes (CD19^+^) was significantly reduced in the group of OSCC patients both pre- and post-therapy. A recent study on a mouse model of oral cancer also showed the accumulation of functionally diverged populations of CD11b^+^ Gr-1^+^ cells in the spleen and tumor tissues (29). The results of the present study demonstrate a reduction in B lymphocytes in both the spleen and blood, but the T-cell population increased in the spleen while it decreased in blood. Further examination of spleen lymphocytes showed a decrease in CD5^+^ B cells in both pre-OSCC and post-OSCC groups. The splenic marginal zone (MZ) B cells, present at the interface between circulation and the white pulp of the spleen, are motile cells with “innate-like” properties, known to bridge the temporal gap until follicular B cells respond in a T-cell-dependent manner; hence, MZ B cells render T-cell-independent responses. In our study, MZ B cells were lower in the pre-OSCC 4-NQO group but significantly higher in the post-OSCC 4-NQO group, suggesting possible suppression of circulating IgM and thus making mice more susceptible (47, 48). γδ T cells differ from other T-cell subtypes by having γ and δ T-cell receptors, and they possess a high antitumor capability. After migrating to the tumor local environment, γδ T cells can lyse cancer cells through the perforin-granzyme pathway (49, 50) and via TRAIL and FasL (51). γδ T cells are also early sources of IFN-γ and TNF-α, which inhibit cancer growth via enhancing antitumor immunity and inhibiting cancer angiogenesis (52, 53). Our results revealed a significant decrease in γδ T cells in the spleen of pre- and post-OSCC models, suggesting a strong immune suppression response due to 4-NQO exposure. Along with T and B lymphocytes, natural killer (NK) cells play a major role in antitumor defense, and alterations in the number, proportion, and function of circulating NK cells have been reported in oral squamous cell carcinoma (54, 55). Natural killer (NK) and natural killer T (NKT) cells play an important role in both innate and adaptive immune systems. It has been reported that compared to levels in the peripheral blood of healthy patients, patients having premalignant oral lesions display an elevated proportion of NK cells; in our study, we also found a significant increase in NK + NKT + DC cells in the 4-NQO-induced pre-OSCC group, suggesting antitumor immune response at an early stage of oncogenesis.

Since we observed 4-NQO-induced immune cell alterations, to demonstrate a direct impact of 4-NQO *in vitro*, cytotoxicity, DNA damage induction, and apoptosis were investigated by using two neoplastic Jurkat T and Daudi B cells. Our findings demonstrated that 4-NQO could markedly reduce the viability of B cells as compared to T cells, and that is due to an increase in DNA damage marked by DSB marker phospho-H2AX protein expression. Previous studies have indicated that SSBs and DSBs are induced *in vitro* and *in vivo* by 4-NQO (1, 56). Furthermore, it is reported that damage to DNA could induce apoptosis or programmed cell death, which is a crucial factor in maintaining tissue homeostasis. Using Annexin-V FACS analysis, our results found that 4-NQO promoted apoptosis in B cells. In conclusion, B-cell line Daudi showed more sensitivity than T-cell line Jurkat toward 4-NQO treatment, and that too even at low concentrations of 4-NQO. Interestingly, animals exposed to 4-NQO were having hyperthermia and a link between mild hyperthermia, p53, and apoptosis in lymphoid cells has been already established (56).

**Figure 7:**
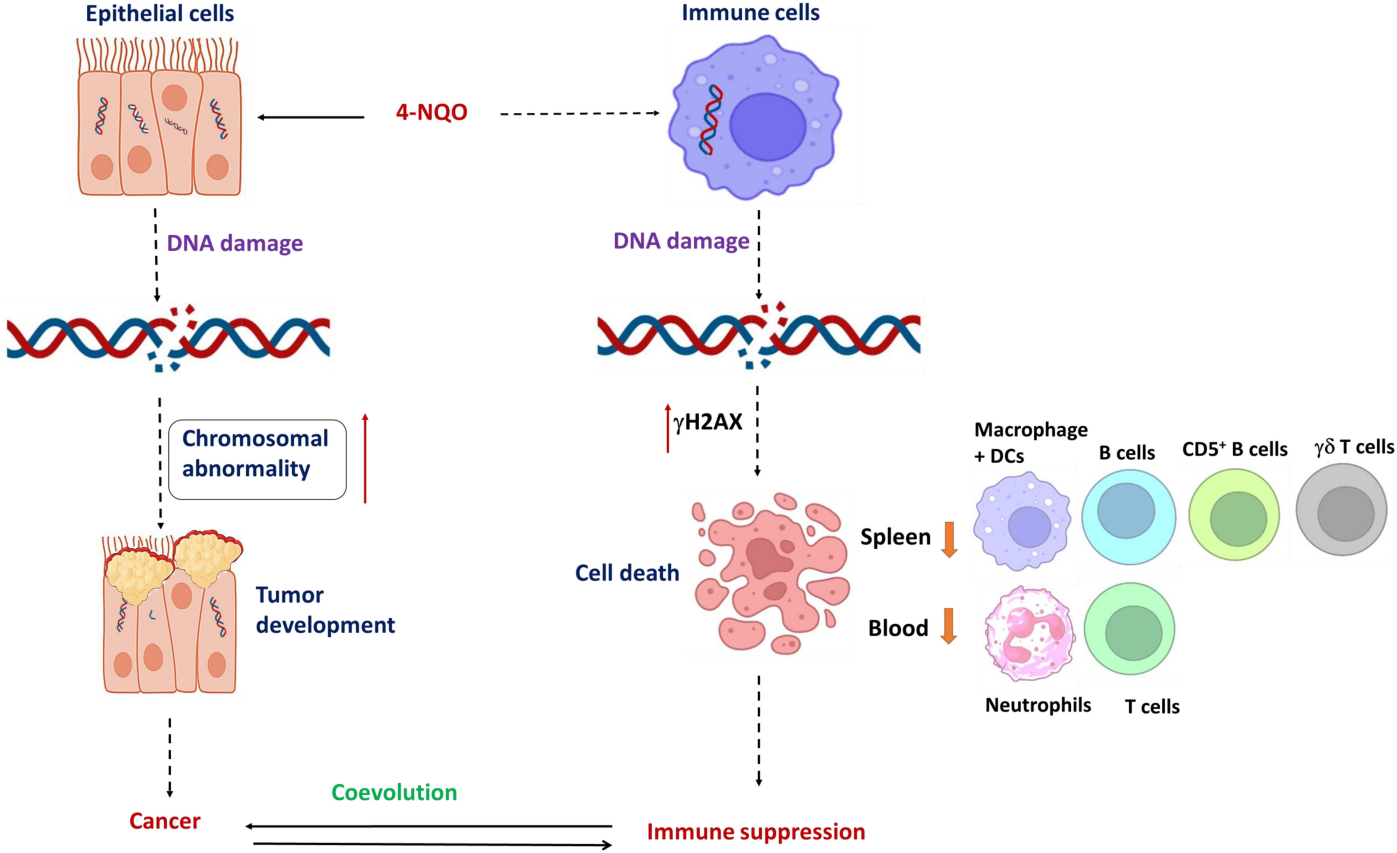
Coevolution of oral oncogenesis and immunosuppression: A graphical representation of 4-NQO-induced carcinogenesis to cause OSCC and 4-NQO-induced cell death of certain immune cells to cause immune suppression. Long-term exposure of epithelial cells to 4-NQO or any other carcinogen; chromosomal abnormalities accumulate to cause cancer, whereas a log-range effect of such carcinogens also causes DNA breaks and cell death in immune cell simultaneously. Mostly the populations of residential B cells, CD5^+^ B cells, γδ T cells, and macrophage + DC populations, and the circulating neutrophils and T cells reduce significantly. Thus, tumor progression and immune suppression occur concurrently and they coevolve further.

In the present study, we found that in addition to 4-NQO’s role in mutagenesis in epithelial cells, it renders selective immunosuppression by varying immune cell representation in both the spleen and blood simultaneously that persists from a precancerous stage to OSCC by causing DNA damage-induced cell death (**Figure 7**). By inducing hyperthermic apoptosis mostly in B lymphocytes and to some extent in T cells, 4-NQO stimulates immunosuppression by mostly reducing their representations. Since the population of γδ T and CD5^+^ B cells, two critical antitumor immune cells, were reduced upon 4-NQO exposure at a precancerous stage, immune suppression seems to be induced at a very early stage of oncogenesis. Thus, carcinogen-induced oncogenesis and immunosuppression coevolve parallelly leading the tumor to thrive. According to IARC, there are 121 carcinogens to humans, and as carcinogens can lead to cancer by damaging the cell’s genome, we believe that similar to 4-NQO, they may also cause immunosuppression. Although the immune checkpoint inhibitors of PD‐1/PD‐L1 and CTLA‐4 appear to be promising therapeutic approaches against various advanced cancers, it appears to be partially effective as only a subset of patients respond to this treatment. Therefore, other immune‐suppressing mechanisms are required to be targeted. Certain immunomodulatory therapeutics that will detoxify 4-NQO could be a promising option. Our study provides new clues to therapeutic strategies in OSCC based on utilizing early host immune response.

## Experimental procedures

### Animals, chemicals and reagents, oligonucleotides, and cell lines

In-house-bred C57BL/6J mice strains (male) of 6–7 weeks were used in the experiments and kept in individually tagged, well-ventilated sterilized cages under standard animal housing conditions. Animals were maintained on normal chow (laboratory animals feed; VNK Nutritional Solution) and normal drinking water before starting the experiments. The experiments were performed under controlled conditions with a 12-h light/dark cycle. The body weight and body temperature were measured before and after NQO treatment. Two cell lines Jurkat (Human T lymphocytes) and Daudi (Human B lymphoma) were cultured in a medium containing RPMI-1640, 10% fetal bovine serum, 2 mmol/L glutamine, and 100 U/ml penicillin and 100 mg/ml streptomycin in a humidified incubator at 37°C with 5% CO_2_. The cell suspension was subcultured in a flask containing fresh medium after every 2 days, and only cells with more than 90% cell viability were used for various assays. 4-Nitroquinoline N-oxide (4-NQO) (Cat#0215592694) and propylene glycol (Cat#151957) were procured from MP Biomedicals India Pvt., Ltd, India. The fetal bovine serum of South American origin (Cat# P30-3302) and Dulbecco’s phosphate-buffered saline without Ca and Mg (Cat# P04-36500) were purchased from PAN Biotech, GmbH, Germany. The cell strainer (nylon mesh; 40 μm; TCR024-1X50NO) and Petri plates (Cat#353147) were obtained from HiMedia and Corning, respectively. Ammonium chloride (NH_4_Cl), sodium bicarbonate (NaHCO_3_), and ethylenediaminetetraacetic acid disodium salt (Na2-EDTA) were purchased from Sigma-Aldrich. The LIVE/DEAD™ Fixable Blue Dead Cell Stain Kit (#2089943) was brought from Thermo Fisher Scientific Inc., MA, and USA. The antibodies which were used in the immune phenotyping and immunofluorescence obtained from different companies are listed below with details (**Table 1**).

**Table 1:** List of antibodies used for immunostaining.

Fluorophore	Marker	Isotype	Company	Cat no.
BV421	CD 21/35	Rat IgG2a, κ	BioLegend	123422
BV510	CD43	Rat DA x LOU IgG2a, κ	BD Horizon	563206
BV 650	CD-TCR	Armenian Hamster IgG_2_, κ	BD Horizon	563993
Alexa Fluor488	CD5	Rat IgG2a, κ	BioLegend	100612
PE	CD23	Rat IgG2a, κ	BioLegend	101607
PECF 594	CD93	Rat IgG_2b_, κ	BD Horizon	563805
PerCp/Cyanine 5.5	CD19	Rat IgG2a, κ	TONBO Biosciences	65-0193-U100
APC	CD161	Mouse IgG2a, κ	TONBO Biosciences	20-5941-U100
Alexa Fluor 700	Gr-1	Rat IgG2b, κ	BioLegend	108422
APC-Cyanine 7	CD11b	Rat IgG2b, κ	TONBO Biosciences	25-0112-U100
PC10	PCNA	Mouse IgG2a, κ	Santa Cruz	sc-56
1C12	p53	Rabbit IgG, κ	CST	2524

### Ethics declaration

Animal experiment was carried out as per the guidelines of the Institutional Animal Ethical Committee, Institute of Life Sciences, Bhubaneswar, India. The protocols were approved with ethical permit numbers ILS/IAEC-89-AH/July-17. All efforts were made to minimize the suffering of animals and ensured the highest ethical and humane standards.

### 4-NQO oral squamous cell carcinoma model

**A** final 100-μg/ml working solution was prepared by dissolving 2 ml 4-NQO stock solution (50 mg/ml in DMSO) in 6 ml propylene glycol and diluted in drinking water with a final volume of 1 L. The experiment was performed in two groups, namely, pre-OSCC (8 weeks) and post-OSCC (24 weeks). Each group contained an experimental cage in which mice were fed with water containing 4-NQO (100 µg/ml), and the corresponding control cage with normal drinking water without 4-NQO. The drinking water (without and with 4-NQO) was replaced weekly. In the pre-OSCC group, mice were administered drinking water for 8 weeks. In the post-OSCC group, the experimental cage mice were administered drinking water containing 100 µg/ml 4-NQO for 8 weeks followed by normal drinking water for 6 weeks and then again exposed to drinking water containing 4-NQO for 8 weeks; however, the control group was provided normal drinking water throughout the experiments. After 8 and 24 weeks of 4-NQO treatment, mice were checked for sickness and tumorous outgrowth in the tongue, following which they were sacrificed, and tongue, blood, and spleen were collected separately for further processing.

### Murine splenocyte isolation

Spleens were collected from the euthanized mice and stored in individually labeled 15-ml Falcon tubes containing 5 ml PBS buffer. For single-cell suspension, the spleens were processed in a cell culture-based sterilized vertical airflow safety cabinet. Spleens were taken out and placed on a Petri dish, and fat was removed with the help of sterilized forceps. A cell strainer having 2 ml of 1× PBS containing 0.1% BSA was placed in a new sterile Petri dish, and the spleen was kept in that strainer. The spleen was meshed gently through the cell strainer into the Petri plates using the plunge end of sterilized syringes. The cell strainer was properly rinsed using 5 ml of 1× PBS with 0.1% BSA, and suspended cells were isolated carefully from the individual Petri plates in a 15-ml Falcon tube. The cell suspension was centrifuged at 380*g* for 8 min at 4°C. After centrifugation, supernatant was discarded, and cells were suspended in 2 ml of 1× RBC lysis buffer, mixed by gentle inversion, and incubated for 5–10 min at room temperature. For each sample, post-incubation suspension volume was maintained up to 13 ml using the same RBC Lysis buffer and centrifuged again. The RBC lysis process was performed two times for the complete lysis of RBCs. After complete RBC lysis, cells were resuspended in 1 ml of 1× PBS+0.1% BSA and centrifuged, and the supernatant was discarded, again followed by resuspension in 1 ml of 1× PBS+0.1% BSA. 10 μl of single-cell suspension was then stained with trypan blue, counted in a hemocytometer, and diluted accordingly.

### Immunophenotyping of splenocytes

Approximately 1 million counted splenic cells were transferred to respective UV-sterilized FACS tubes with a final volume of 1 ml. The cells were washed with 1 ml of 1× PBS+0.1% BSA followed by antibody staining. The fluorochrome-conjugated antibodies were diluted in 1× PBS–0.1% BSA with a final concentration of 18 μg/ml. Each antibody was added individually in all the samples along with single-colored control and incubated for 30 min in the dark at room temperature for optimal staining. After incubation, cells were washed with 1× PBS+0.1% BSA, and live-dead staining was performed using Fixable Blue Dead Cell Stain Kit. At first, 1 ml of 1× PBS was added to each sample followed by the addition of 1 μl solution A and incubation for 15–20 min in the dark at room temperature. After incubation, 1 ml of 1× PBS+0.1% BSA was added and then centrifuged at 380*g* for 8 min at 4°C. The cells were taken up in 500 μl of 1× PBS+0.1% BSA. Then, the samples were acquired in BD LSRFortessa Flow Cytometer (BD Biosciences) and after the acquisition, data were analyzed in FlowJo software.

### Immunophenotyping of white blood cells

Approximately 150μl of blood was collected in a sterilized microcentrifuge tube containing 10% K2-EDTA anticoagulant by cardiac puncture using a 2-ml syringe and kept on ice. The blood cells were transferred into 15-ml tubes and resuspended with 2 ml of 1× RBC lysis buffer and incubated for 2–3 min, followed by neutralization by adding 1 ml of 1× PBS+0.1% BSA. The rest of the cells were collected by centrifugation at 380*g* for 8 min at 4°C, and the cells were again washed with 1 ml of 1× PBS+0.1% BSA. Finally, cells were resuspended in 500 μl of 1× PBS+0.1% BSA for antibody staining. The Fixable Blue Dead Cell Stain Kit was used to differentiate between live and dead cells. Cells were then suspended in 1 ml of 1× PBS+0.1% BSA and centrifuged at 380*g* for 8 min at 4°C, and the cell pellet was resuspended in 500 µl of 1× PBS+0.1% BSA. For staining, the fluorochrome-conjugated antibodies were added and incubated for 30 min in the dark at room temperature, followed by 1× PBS+0.1% BSA washing and further resuspension in the same. The samples were then collected using a BD LSRFortessa Flow Cytometer (BD Biosciences), and FlowJo software was used to examine the data.

### H&E staining

The mice were sacrificed, and tongues were isolated and fixed in 4% formalin and kept at room temperature until experimental analysis. Fixed tongues were sectioned in LEICA RM2125 RTS microtome with 5-µm thickness, dewaxed in xylene, and rehydrated with gradually decreasing concentrations of ethanol (100%, 95%, and 75%) and distilled water. Sections were stained with hematoxylin for 3 to 5 min, followed by counterstaining with eosin for 2 min with intermediate washing with tap water to confirm the histological diagnosis. The slides were dehydrated with gradually increasing concentrations of ethanol (75%, 95%, and 100%) and then mounted using DPX mounting medium, and images were acquired under a LEICA DM500 microscope with 10× and 40× magnification.

### Immunohistochemistry

Separate tongues were used for various immunohistochemistry. In brief, the tongue sections were dewaxed in xylene and hydrated with decreasing gradient ethanol, followed by exposure to 3% hydrogen peroxide solution for 10 min to remove endogenous peroxidase. The antigen retrieval step was performed by heating in a steamer at 95°C for 20 min in the antigen retrieval buffer. After cooling at room temperature, the sections were treated with anti-p53 mouse monoclonal antibody (1:1,000 dilution) and incubated at 4°C overnight in a moist chamber. The sections were then treated with biotinylated universal antibody (anti-rabbit/mouse IgG) and incubated at 37°C for 25 min. Finally, 3,3′diaminobenzidine (DAB) chromogen and hematoxylin were used to visualize immunoreactivity and for counterstaining, respectively. Images were acquired under the LEICA MD500 microscope with 40× magnification.

### Immunofluorescence

For immunofluorescence staining, tongue-sectioned slides were dewaxed in xylene and hydrated with decreasing amount of gradient ethanol, followed by antigen retrieval via heating in a steamer at 95°C for 20 min. Slides were then incubated for 30 min in 1% BSA for blocking the non-specific binding of antibodies. After blocking, anti-PCNA mouse monoclonal primary antibody (PC10) was applied on a hydrophobic layer around the tongue sections and incubated at 4°C overnight in a humidified chamber. Next day, Alexa Fluor 488 anti-mouse IgG antibody (1:5,000) was used for secondary staining and samples were incubated in a humidified chamber for 45 min. Post-incubation slides were mounted using DAPI containing the mounting agent, and images were acquired using a confocal microscope (Leica, STED).

### Cell viability assay

Cell viability was evaluated by MTS assay, which is a colorimetric quantification of viable cells, based on the principle of reduction of MTS tetrazolium compound by NAD(P)H-dependent dehydrogenase enzymes present in the metabolically active cells viable cells, to produce a colored formazan product soluble in cell culture media. Jurkat and Daudi cells were seeded in a 48-well plate and were exposed to different concentrations of 4-NQO. Following 48 h treatment of 4-NQO, 20 μL of MTS reagent was added into each well and incubated at 37°C for 3–4 h. Quantification was done by measuring absorbance at 490 nm with the ELISA plate reader. All experiments were repeated three times. A trypan blue assay was also carried out to assess the effect of 4-NQO on the viability of Jurkat and Daudi cells. Briefly, Jurkat and Daudi cells were cultured in six-well plates in duplicates and treated with 4-NQO for 48 h. After 48 h, cells were counted using trypan blue staining and plotted. In both assays, untreated cells were taken as control groups.

### Protein isolation and Western blot

Jurkat and Daudi cells were treated with 0.5, 1, 1.5, and 2 μM 4-NQO for 48 h and analyzed for γH2AX expression. The cells were lysed in RIPA buffer by centrifugation at 12,000 rpm for 20 min and the whole-cell lysate (supernatant) was transferred to a clean Eppendorf tube, and the protein concentration was determined using the Bradford method. After that, aliquots of sample lysates with 40 μg of total protein were separated by SDS-polyacrylamide gel electrophoresis on 12% Tris-glycine gels and transferred onto 0.4-μm polyvinylidene difluoride membranes (PVDF). Blots were then blocked with 5% BSA, probed with a primary antibody specific for γH2AX (cat. no. 9718; Cell Signaling Technologies) and β-actin (sc-47778, Santa Cruz Biotechnology), followed by incubation with secondary anti-rabbit (A0545, Sigma) and anti-mouse (A5278, Sigma) IgG horseradish peroxidase antibodies, respectively, and then visualized using ECL (enhanced chemiluminescence) (GE Healthcare, Amersham). The band intensity was quantified and normalized with the corresponding endogenous control β-actin using ImageLab software and plotted and presented as a histogram.

### Confocal analysis

Confocal laser scanning microscopy (CLSM) experiments were performed to detect γH2AX distribution in the nucleus of Jurkat and Daudi cells, following 4-NQO treatment. Microscopic glass coverslips were coated with poly-L-lysine and placed in six-well plates. Cells were allowed to attach and treated with 1.5 and 2 μM 4-NQO. Cells were then fixed with 4% paraformaldehyde for 15 min, permeabilized with 0.1% Triton X-100 for 5 min, and blocked with 5% BSA for 1 h at 37°C. Subsequently, the cells were incubated with primary monoclonal antibody anti-γH2AX (1:100 dilution, cat. no. 9718; Cell Signaling Technology Inc.) overnight at 4°C, and with Alexa Fluor 488 (1:500 dilution, cat. no. A11017, Invitrogen) at room temperature for 1 h. Following washing with 1× PBS, DAPI (1.5 µg/ml) was added to counterstain the nuclei, and γH2AX foci were detected with a confocal microscope.

### *In vitro* assessment of apoptosis (Annexin V/propidium iodide assay)

Cell apoptosis was determined by Annexin V and PI staining. Briefly, Jurkat and Daudi cells were cultured in the presence and absence of 4-NQO for 48 h. Cells (10^6^/sample) were pelleted down, washed with PBS, centrifuged at 200*g* for 5 min, and resuspended in 1× binding buffer. Cells were then stained with Annexin V and incubated for 15 min at RT. After incubation, cells were centrifuged and resuspended again in 1× binding buffer. Immediately prior to analysis by flow cytometry, PI was added to each sample. The fluorescence signals were measured by BD LSR Fortessa and analyzed using FlowJo software.

### Statistical analyses

Statistical analyses of data sets derived from all experiments were carried out using GraphPad Prism 8.0 and Microsoft Excel. Statistical significance was calculated based on two-way or one-way ANOVA, with Tukey’s *post-hoc* multiple-comparison test. Data are presented as mean ± standard error (SEM) and statistical significance as ns = non-significant, *P-value <0.05, **P-value <0.01, and ***P-value <0.001.

## Data availability statement

The original contributions presented in the study are included in the article/**Supplementary Material**. Further inquiries can be directed to the corresponding author.

## Ethics statement

Ethical approval was not required for the studies on humans in accordance with the local legislation and institutional requirements because only commercially available established cell lines were used. The animal study was approved by ILS Animal Ethics Board (ILS/IAEC-89-AH/July-17). The study was conducted in accordance with the local legislation and institutional requirements.

## Author contributions

SS: Data curation, Formal Analysis, Investigation, Methodology, Resources, Validation, Writing – original draft, Writing – review & editing. ST: Data curation, Formal Analysis, Investigation, Methodology, Resources, Validation, Writing – original draft, Writing – review & editing, Software. DP: Data curation, Formal Analysis, Investigation, Methodology, Resources, Validation, Writing – original draft, Writing – review & editing, Software. BU: Methodology, Resources, Writing – original draft, Writing – review & editing, Investigation. AD: Methodology, Resources, Writing – original draft, Writing – review & editing. PK: Methodology, Resources, Writing – original draft, Writing – review & editing. IS: Methodology, Resources, Writing – original draft, Writing – review & editing, Investigation. NA: Conceptualization, Funding acquisition, Project administration, Supervision, Validation, Visualization, Writing – original draft, Writing – review & editing, Formal Analysis, Investigation, Resources.
