# Supplementary material for: 4-nitroquinoline 1-oxide induces immune cells death to onset early immunosuppression during oral squamous cell carcinoma development
Source: Front Immunol. 2023 Oct 23;14:1274519. doi: 10.3389/fimmu.2023.1274519 (PMC10626482; doi:10.3389/fimmu.2023.1274519)
Supplement: Supplementary file 1 [file DataSheet_1.pdf]

Supporting Informations

4-Nitroquinoline 1-oxide induces immune-cells death to onset early immunosuppression during Oral squamous cell carcinoma development

Satya Ranjan Sahu[1,2,+], Shweta Thakur[1,+], Doureradjou Peroumal[1,+], Bhabasha Gyanadeep Utkalaja[1,2], Abinash Dutta[1], Premlata Kumari[1,2], Ipsita Subhadarsini[1,2], and Narottam Acharya[1,*]

[1]Laboratory of Genomic Instability and Diseases, Department of Infectious Disease Biology, Institute of Life Sciences, Bhubaneswar-751023, India.

[2]Regional Center of Biotechnology, Faridabad, India

*__Correspondence to__:

Narottam Acharya, Phone: 91-674-2304278, Fax: 91-674-230 0728

E-mail: narottam_acharya@ils.res.in; narottam74@gmail.com

+ contributed equally

Running title: Altered immune-cell profiling by 4-NQO

Keywords: 4-NQO, DNA damage response, cell death, PCNA, p53, Immune cell, T cells, B cells, Oral squamous cell carcinoma, Cancer immunology,

Abbreviations: 4-NQO: 4-nitroquinoline 1-oxide; OSCC: Oral squamous cell carcinoma; EGFR: Epidermal growth factor receptor; NK: Natural killer; NKT: Natural killer T cells; RAGE: Receptor for advanced glycation end products; MUC1: Mucin 1; FACS: flow cytometry.

Fig. S1: A gating strategy to immunophenotyping splenocytes (i) and blood cells (ii)

Fig. S2: Representative photograph of individual spleens of mice exposed to 4-NQO (i) and their length measurements (ii).

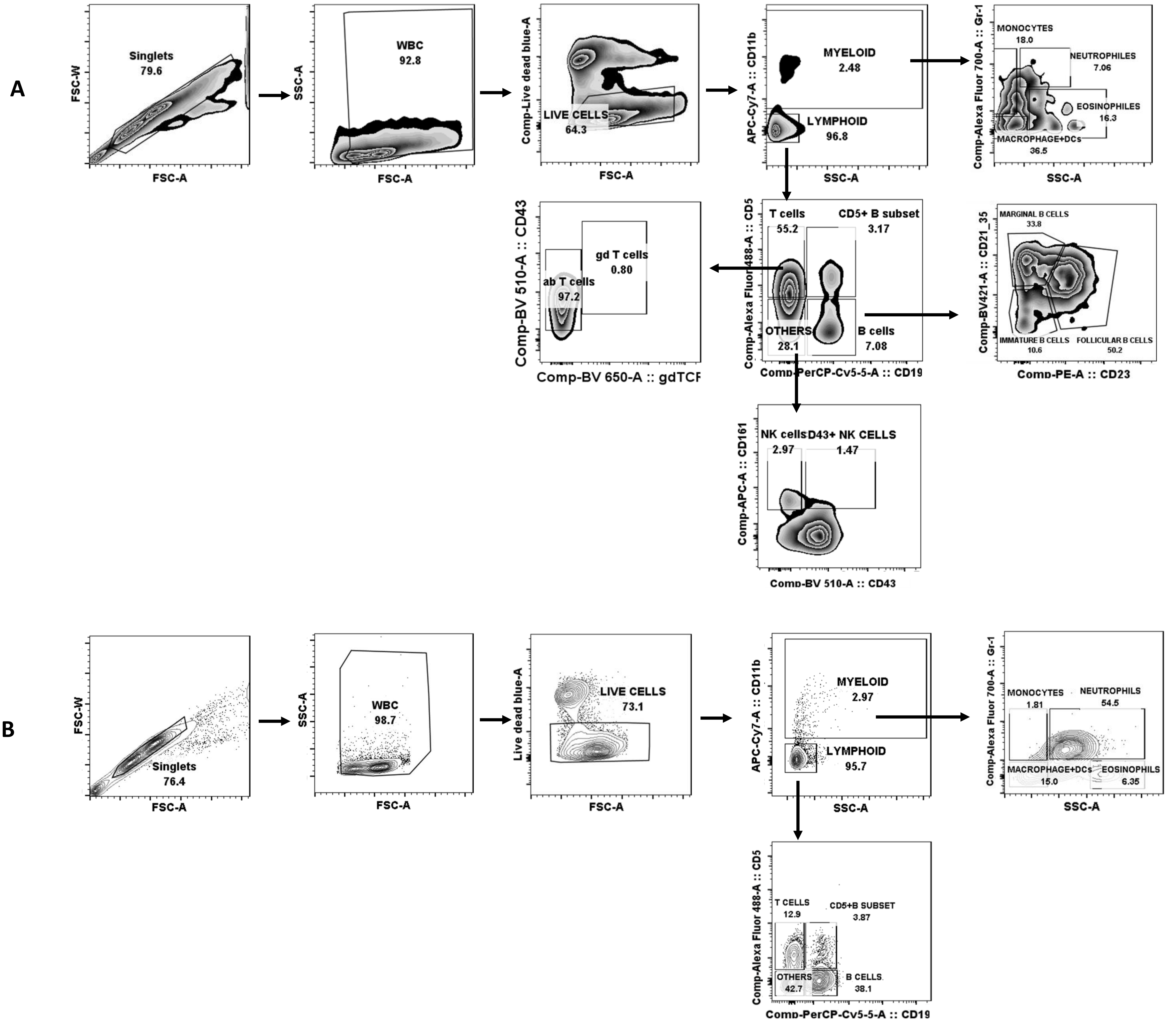

Supplementary Fig. 1

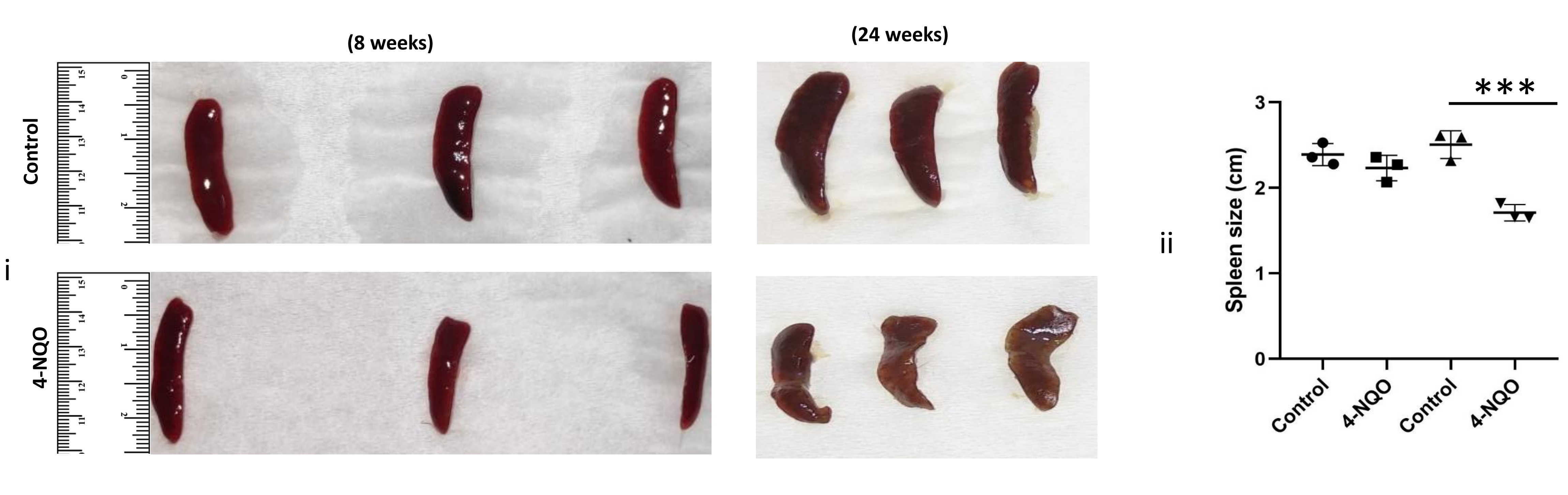

Supplementary Fig. 2
